# MET-targeting antibody (emibetuzumab) and kinase inhibitor (merestinib) as single agent or in combination in a cancer model bearing *MET* exon 14 skipping

**DOI:** 10.1007/s10637-017-0545-x

**Published:** 2017-11-29

**Authors:** S. Betty Yan, Suzane L. Um, Victoria L. Peek, Jennifer R. Stephens, Wei Zeng, Bruce W. Konicek, Ling Liu, Jason R. Manro, Volker Wacheck, Richard A. Walgren

**Affiliations:** Lilly Research Laboratories, Eli Lilly and Company, DC0522, 307 E. McCarty Street, Indianapolis, IN 46285 USA

**Keywords:** Merestinib, Emibetuzumab, MET exon 14 skipping, MET kinase inhibitor, MET antibody, LY2801653, LY2875358

## Abstract

*Purpose* Approximately 3% of lung cancer bears mutations leading to MET exon 14 skipping, an oncogenic driver which is further evidenced by case reports of patient response to MET kinase inhibitor treatment. Approximately 15% of tumors harboring MET exon14 skipping have concurrent *MET* amplification. *Experimental Design* Merestinib is a type II MET kinase inhibitor. Emibetuzumab, a bivalent anti-MET antibody, internalizes MET receptor. Each single agent and the combination were evaluated in the Hs746t gastric cancer line bearing MET exon14 skipping and *MET* amplification. *Results* Merestinib inhibited Hs746t cell proliferation (IC_50_=34 nM) and totally eliminated pMET at 100nM. Emibetuzumab showed little anti-proliferative activity against Hs746t cells (IC_50_>100nM), did not reduce pMET, and slightly reduced cell surface MET. In the Hs746t xenograft model, dose dependent differences in durability of response were seen with merestinib including durable tumor regression (91.8%) at 12 mg/kg qd. Emibetuzumab treatment (10mg/kg qw) provided transient tumor regression (37.7%), but tumors re-grew while on treatment. Concurrent combination of merestinib (6 mg/kg qd) and emibetuzumab resulted in 85% tumor regression, while a sequential combination (initiating merestinib first) resulted in longer duration of treatment response. *Conclusions* Data in this study support a clinical evaluation of merestinib in patients with MET exon 14 skipping (NCT02920996). As a type II MET kinase inhibitor, merestinib may provide a therapeutic option to treatment naïve patients or to patients who progress on type I MET inhibitor treatment. Data also support clinical evaluation of the sequential combination of merestinib with emibetuzumab when patients progress on single agent merestinib.

**Electronic supplementary material:**

The online version of this article (10.1007/s10637-017-0545-x) contains supplementary material, which is available to authorized users.

## Introduction

The HGF/MET signaling pathway regulates a wide variety of normal cellular functions that can be subverted to support neoplasia, including cell proliferation, survival, apoptosis, scattering and motility, invasion, and angiogenesis [1]. Over-expression of the MET receptor or its ligand HGF, *MET* amplification or gain-of-function mutation in MET are the various mechanisms for which MET pathway dysregulation is implicated in cancer development and progression. Mutations that lead to the skipping of exon 14 in *MET* have been detected in several cancers and are most prevalent in lung cancer at an incidence of about 3% in both Western and Asian countries [2–10]. Importantly, Y1003 which resides within exon 14 of MET, constitutes the docking site for the E3 ubiquitin-protein ligase CBL (Casitas B-lineage lymphoma). Thus, mutations leading to exon 14 skipping result in loss of Y1003, and subsequently block CBL-mediated MET degradation, leading to sustained MET oncogenic signaling [11]. MET exon 14 skipping acting as an oncogenic driver is further evidenced by case reports of patient response to treatment with MET-targeting kinase inhibitor [12, 13]. Additional clinical findings also reported the existence of a point mutation at residue Y1003, which exhibits the MET exon 14 skipping phenotype [8]. Occurrence of MET exon 14 skipping in lung cancer is mutually exclusive from other oncogenic driver mutations such as EGFR activating mutations, ALK fusion, and ROS1 fusion [8, 9].

Merestinib (LY2801653) is an orally bioavailable, type II MET kinase inhibitor, with a slow binding off-rate, and has demonstrated anti-tumor and anti-angiogenic activity in several *MET* amplified and MET autocrine xenograft tumor models [14, 15]. Emibetuzumab (LY2875358) is a bivalent anti-MET IgG_4_ antibody that blocks HGF binding to the MET receptor as well as internalizes MET, leading to receptor degradation and inhibition of ligand-dependent signaling [16]. The Hs746t human gastric cancer line contains a G > T splicing mutation at intron 14 + 1 of *MET*, resulting in the skipping of exon 14 in the mature mRNA [17]. This cancer line also has concurrent *MET* amplification [18, 19]. Thus, the Hs746t cancer model resembles approximately 15% of the tumors reported in the literature bearing MET exon 14 skipping that have concurrent *MET* amplification [8, 9]. Merestinib was shown previously to inhibit the proliferation of Hs746t cells in vitro [14] with an IC_50_ of 34 nM. In this study, merestinib alone or in combination with emibetuzumab was evaluated further in vitro and in vivo in mice bearing Hs746t xenograft tumors.

## Materials and methods

Hs746t and MKN45 cells were obtained from ATCC (Manassas, VA) and Japan Health Sciences Foundation, Health Science Research Resources Bank (Osaka, Japan), respectively. PF04217903 and savolitinib were obtained from Selleck Chemicals (Houston, TX) and ChemieTek (Indianapolis, IN), respectively. Humanized one-armed OA-5D5 MET antibody was expressed in CHO cells [16].

### Western blotting

Hs746t cells were maintained in DMEM Medium with L-glutamine and 10% FBS. MKN45 cells were maintained in RPMI 1640 Medium with L-glutamine, 10% FBS, and sodium pyruvate. Cells were grown at 37 °C with 5% CO_2_, seeded into 6-well plates at 10^6^cells/well, and incubated overnight. Testing compound was added and incubated for 24 h, and cells were lysed with RIPA buffer containing protease and phosphatase inhibitors. Protein concentrations of cell lysates were measured with the DC Protein Assay (Bio-Rad, Hercules, CA). Lysates were electrophoresed on Novex 4–20% Tris Glycine gels (Invitrogen, Carlsbad, CA), and transferred onto PVDF membranes. The blots were probed with antibodies obtained from Cell Signaling (Danvers, MA) for: MET (clone D1C2) (Cat #8198), p-MET (Y1234/1235) (clone D26) (Cat #3077), p-MET (Y1003) (clone 13D11) (Cat #3135), EGFR (clone D38B1) (Cat #4267), pEGFR (Y1068) (clone D7A5) (Cat #3777), ERK1/2 (clone L34F12) (Cat #4696), pERK1/2 (T202/Y204) (Cat #9101), AKT (clone 40D4) (Cat #2920), pAKT (S473) (clone D9E) (Cat #4060), Gab1 (Cat #3232), pGAB (Y627) (clone C32H2) (cat #3233); from BD Biosciences (San Jose, CA): EIF4E (clone 87/EIF-4E) (Cat #610270); and from Abcam (Cambridge, MA): p-EIF4E (S209) (clone EP2151Y) (Cat #ab76256). β-Actin (clone AC-15) (Sigma Cat #A5441) (St. Louis, MO) was used as a loading control. After incubating with HRP-linked secondary antibodies, blots were developed with chemiluminescent substrate and imaged on an ImageQuant LAS 4000 imager (GE Healthcare).

### Cell proliferation assay

Hs746t cells were seeded onto poly-D-lysine, 96-well plates, at 3000 cells/well and allowed to attach overnight in a 37 °C incubator with 5% CO_2_. Testing compounds were serially diluted 1:3 and added to the cells in triplicate. After 120 h, cell viability was measured with the CellTiter-Glo Luminescent Cell Viability Assay (Promega, Madison, WI). The data was analyzed with GraphPad Prism v6 software. The assay was performed twice for each compound in separate experiments.

### In vivo mouse studies

All animal studies were performed in accordance with the International Association for Assessment and Accreditation of Laboratory Animal Care institutional guidelines. All in vivo experimental protocols were approved by the Eli Lilly and Company Animal Care and Use Committee. Female athymic nude mice were obtained from Envigo (Indianapolis, Indiana). Hs746t cells were expanded in culture, harvested, and washed in HBSS. Approximately 5 × 10^6^ cells in HBSS were implanted subcutaneously into the hind flank of each animal. Merestinib was formulated as a solution in 10% PEG 400/90% (20% Captisol in water) [PEG-400 from Fisher Scientific Cat#P167–1; Captisol from Cydex Pharmaceuticals (Lenexa, Kansas)]. Crizotinib was formulated as a solution in 10% acacia/0.05% antifoam. Emibetuzumab was diluted in PBS. Animals were removed from the study when tumor burden exceeded 2000 mm^3^.

### Statistical analyses

Tumor volumes and body weight were measured bi-weekly. Statistical analysis was performed on the last study day when no more than half of the animals in the vehicle group were removed from the study due to tumor burden.

Tumor volume was transformed to the log scale to equalize variance across time and treatment groups. The log volume data were analyzed with a two-way repeated measures analysis of variance by time and treatment using the MIXED procedures in SAS software (Version 9.3).

The correlation model for the repeated measures was spatial power. Treated groups were compared to the control group at each time point. The MIXED procedure was also used separately for each treatment group to calculate adjusted means and standard errors at each time point. Both analyses account for the autocorrelation within each animal and the loss of data that occurred when animals were removed or lost before the end of the study. The adjusted means and standard errors were plotted for each treatment group versus time.

## Results

### In vitro studies

Merestinib was previously reported to inhibit Hs746t cell proliferation with IC_50_ = 34 nM [14], and this observation was reproduced in this study (Table 1) with an IC_50_ of 33.4 nM. The anti-proliferative effect of several MET kinase inhibitors was compared and IC_50_ values were found to be similar to merestinib (Table 1). PF04217903 is the most highly specific MET kinase inhibitor evaluated. Emibetuzumab, as well as the one-arm MET antibody OA-5D5, showed little in vitro anti-proliferative activity (Table 1).

**Table 1:** In vitro activity of MET inhibitors on the proliferation of Hs746t tumor cells bearing *MET* exon 14 skipping and *MET* amplification

Compound	IC_50_ (nM)
Merestinib (LY2801653)	33.4
Crizotinib	40.4
Cabozantinib	41.8
Emibetuzumab (LY2875358)	>100
OA-5D5	>200

**Fig. 1:**
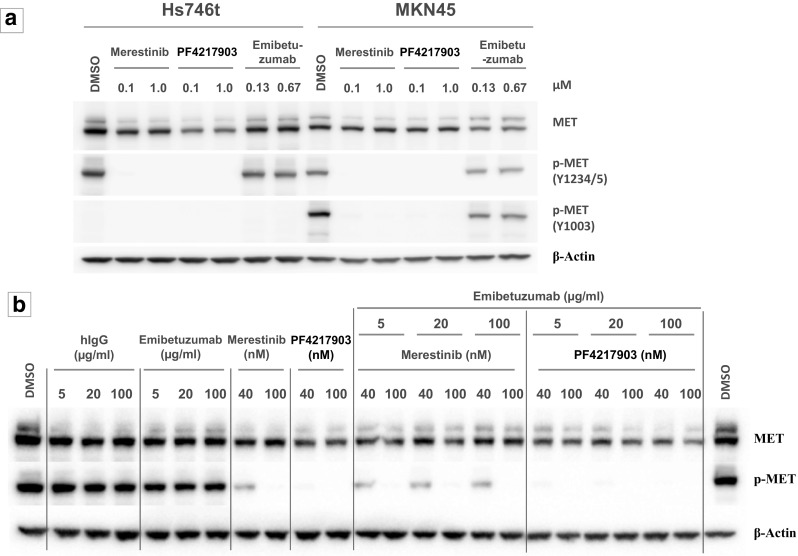
In vitro effect of MET inhibitors on expression of total MET, pMET (Y1234/1235), and pMET (Y1003). **a** Western blot analysis of Hs746t (exon 14 skipping and *MET* amplification) and MKN45 (*MET* amplification) cells treated for 24 h with merestinib, PF04217903, or emibetuzumab at indicated concentrations. Blots were probed with antibodies to total MET, phospho-MET (Y1234/1235), and phospho-MET (Y1003). **b** Western blot analysis of Hs746t cells treated for 24 h with single agent MET inhibitors (emibetuzumab, merestinib, or PF04217903) or hIgG_4_ at the indicated concentrations or combination of emibetuzumab with either merestinib or PF04217903. Blots were probed with antibodies to total MET, and phospho-MET (Y1003)

The effects of merestinib and emibetuzumab on total MET and pMET expression in Hs746t cells were also evaluated, with MKN45 cells serving as a comparator. The Hs746t human gastric cancer line contains a homozygous G > T genomic splicing mutation at intron 14 + 1 of *MET*, resulting in the skipping of exon 14 in the mature mRNA [17]. This cancer line also has concurrent *MET* amplification [18, 19]. *MET* is highly amplified in MKN45 gastric cancer cells, but with no MET exon 14 skipping. Hs746t cells treated with 100 nM merestinib or PF04217903 abrogated pMET (Y1234/Y1235) (Fig. 1a, b). Residues pY1234/1235 are located outside of exon 14, whereas pY1003 resides within exon 14 and is the key residue for CBL docking. As expected, pY1003 expression was absent in Hs746t cells, but was present in MKN45 cells as determined by western blot (Fig. 1a). Merestinib or PF04217903 at 100 nM also totally eliminated phospho-Y1003 in MKN45 cells (Fig. 1a). Emibetuzumab previously was shown to reduce total MET and pMET in MKN45 cells by about 40–50% [16], and those results were reproduced here (Fig. 1a). The reduction of total MET with emibetuzumab treatment in Hs746t cells was less than that in MKN45 cells, at about 10–20%, based on western blot (Fig. 1a) and FACS analysis (Supplementary material, Fig. S1a). Reduction of pMET by emibetuzumab in Hs746t and MKN45 cells was also considerably less than that of the kinase inhibitor merestinib or PF04217903 (Fig. 1b).

**Fig. 2:**
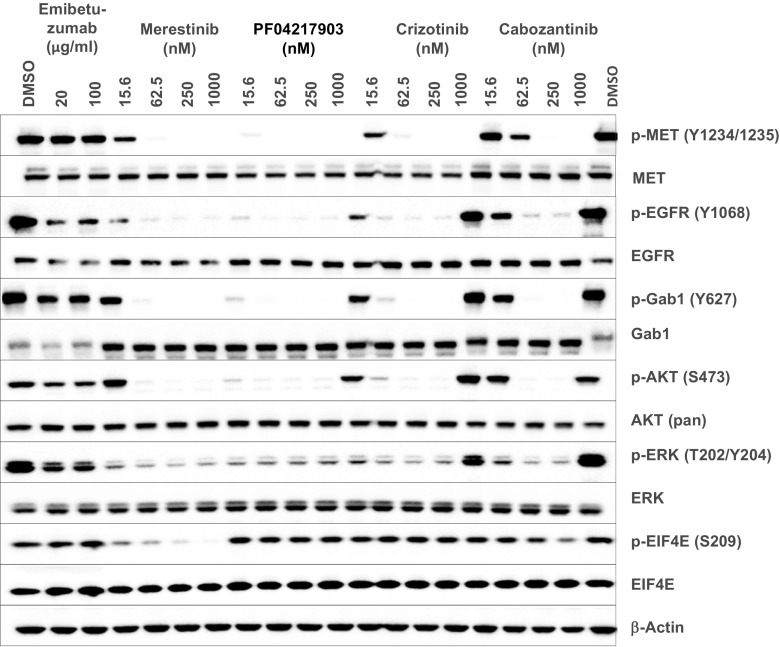
MET kinase inhibitors but not emibetuzumab inhibited MET signaling pathway in Hs746t cells in vitro by western blot analysis. Hs746t cells were treated for 24 h with 133 nM (20 μg/ml) or 667 nM (100 μg/ml) emibetuzumab or MET kinase inhibitors (merestinib, PF04217903, crizotinib, cabozantinib) at 15.6, 62.5, 250 or 1000 nM

MET-targeting kinase inhibitors, both highly specific (PF04217903) and non-specific (merestinib, crizotinib, cabozantinib) totally inhibited pMET in Hs746t cells, leading to a reduction of pAKT, pERK (Fig. 2) and the phosphorylation of the MET signaling adaptor protein GAB1 [1] (Fig. 2). MET-targeting antibody, emibetuzumab did not exhibit these activities (Fig. 2). Interestingly, merestinib-, PF04217903-, crizotinib- or cabozantinib-mediated reduction of pMET also led to reduction of pEGFR, suggesting potential cross-activation between MET and EGFR in Hs746t cells. Merestinib was previously reported to inhibit MKNK1/2 with subsequent reduced phosphorylation of EIF4E (S209) [14]. EIF4E is phosphorylated by MKNK1/2, which are downstream of the RAS/RAF/MEK/ERK and p38 signaling pathways, and is a major cap-binding protein involved in the regulation of mRNA translation in normal and cancer development [20]. Merestinib, but not PF04217903 or crizotinib, completely blocked p-EIF4E in Hs746t cells (Fig. 2). Cabozantinib partially inhibited p-EIF4E, which suggests it may also have direct, albeit weak inhibitory activity on MKNK1/2.

The combination of emibetuzumab with either merestinib or PF04217903 did not result in further reduction of pMET beyond what was achieved with the respective single agent MET kinase inhibitor (Fig. 1b). However, an enhanced reduction of total MET in the combination treatments was observed by western blot analysis (Fig. 1b). The reduction in cell surface total MET upon treatment with the combination of merestinib with emibetuzumab was also confirmed by FACS analysis (Supplementary material, Fig. S1b). Total MET reduction observed with the combination treatment plateaued at about 50% by FACS analysis, with no further reduction with increasing concentration of merestinib.

#### In vivo studies

**Fig. 3:**
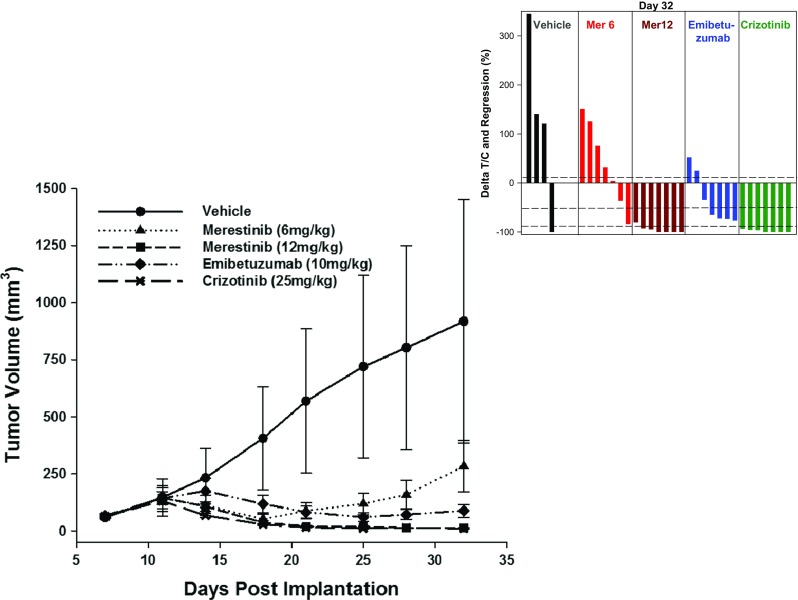
In vivo single agent anti-tumor effect of merestinib, emibetuzumab, and crizotinib in the Hs746t xenograft model. Approximately 5 million cells were implanted in female athymic nude mice (*n* = 7 per group). Vehicle (), merestinib at 6 mg/kg (suboptimal dose – insufficient target coverage for 24 h) () or 12 mg/kg (optimal dose) () was dosed once daily orally, emibetuzumab at 10 mg/kg () was dosed once weekly intraperitoneally (IP), or crizotinib at 25 mg/kg () was dosed once daily orally. Dosing of inhibitors began on day 11, when tumors were of average size of 150 mm^3^, and continued for 21 days. The waterfall plot shows the response of individual animals to treatment on day 32. Missing bars are due to animals removed early from the study because of tumor burden. In the waterfall plot, the three horizontal dotted lines represent the three cut points for: Progressive Disease as ∆T/C > 10%; Stable Disease as ∆T/C ≤ 10% to regression <−50%; Partial Response as regression ≥ − 50% and tumor volume > 14 mm^3^; Complete Response as tumor volume ≤ 14 mm^3^. Statistical analysis comparing tumor volumes of treatment groups to vehicle control group () was performed on Day 32, where at least half of the animals (4/7) in the vehicle group still remained on study. Dosing for merestinib (12 mg/kg) and crizotinib continued to Day 64 and tumor growth monitored for another 5 weeks post-treatment (data not shown)

To examine if merestinib or emibetuzumab reduced tumor growth in vivo, Hs746t cells were implanted in athymic nude mice. Crizotinib was used as a comparator, as most of the case reports of cancer patients with MET exon 14 skipping treated with MET kinase inhibitor were with crizotinib [12, 13]. Treatment with single agent merestinib, emibetuzumab and crizotinib was initiated when average tumor size reached 150mm^3^ (Day 11) (Fig. 3). Merestinib (12 mg/kg once daily) treatment resulted in 91.8% tumor regression (*p* < 0.001) after 21 days of dosing (Day 32). As expected, merestinib administered at a suboptimal dose of 6 mg/kg once daily (insufficient to cover the MET target), resulted in only transient tumor regression, followed by tumor re-growth while on treatment. After 21 days of dosing, the T/C was 18.3% (*p* = 0.033) as compared to the vehicle group. Crizotinib treatment, at 25 mg/kg once daily, resulted in 92.9% tumor regression (*p* < 0.001) after 21 days of dosing. There was no significant difference in the antitumor effect of merestinib (12 mg/kg dose) and crizotinib (*p* = 0.76). Emibetuzumab (10 mg/kg once weekly dosing) showed delayed tumor regression relative to either single agent kinase inhibitor. Tumor regression was transient and tumors re-grew while on treatment. Tumor regression was 37.7% in the emibetuzumab group (p < 0.001) at dosing completion (Day 32) (Fig. 3). In this study, no tumor outgrowth (acquired resistance) was observed in either merestinib (12 mg/kg) or crizotinib (25 mg/kg) treated cohorts with continuous dosing for 64 days (data not shown). Furthermore, the tumor regression observed in these two cohorts was durable with no evidence of tumor re-growth 5 weeks after treatment termination (data not shown). All treatments were well tolerated as monitored by body weight assessment.

**Fig. 4:**
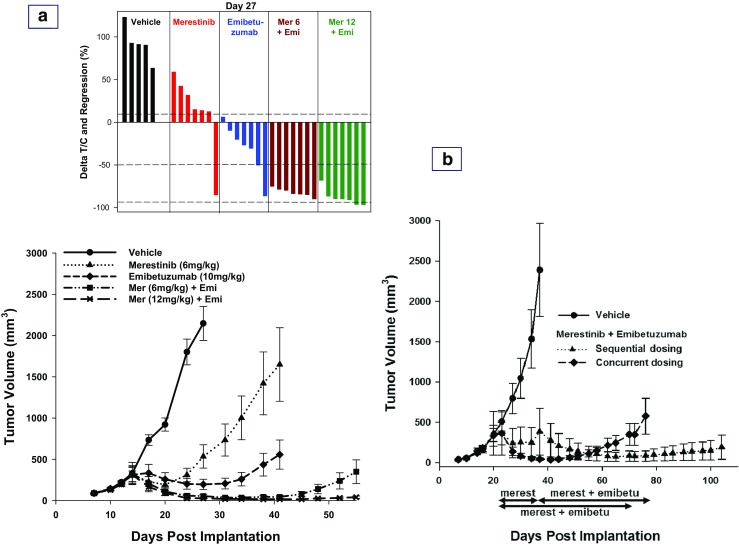
In vivo anti-tumor effect of the combination of merestinib and emibetuzumab in the Hs746t xenograft model. **a** Approximately 5 million cells were implanted in female athymic nude mice (*n* = 7 per group). Dosing of inhibitors began on day 14, when tumors reached average size 350 mm^3^, and continued for 28 days. As single agent, merestinib at 6 mg/kg (suboptimal dose – insufficient target coverage for 24 h) () was dosed once daily orally, and emibetuzumab at 10 mg/kg () was dosed once weekly intraperitoneally (IP). In combination, emibetuzumab at 10 mg/kg was dosed once weekly intraperitoneally (IP) concurrently with either merestinib at 6 mg/kg (suboptimal dose) () or merestinib at 12 mg/kg (optimal dose) () once daily orally. The waterfall plot shows the response of individual animals to treatment on day 27, when tumor volume analysis in comparison to the vehicle control group was performed. Missing bars are due to animals removed early from the study because of tumor burden. In the waterfall plot, the three horizontal dotted lines represent the three cut points for: Progressive Disease as ∆T/C > 10%; Stable Disease as ∆T/C ≤ 10% to regression <−50%; Partial Response as regression ≥ − 50% and tumor volume > 14 mm^3^; Complete Response as tumor volume ≤ 14 mm^3^. **b** Comparison of sequential and concurrent combination of merestinib (6 mg/kg once daily) with emibetuzumab (10 mg/kg once weekly). Approximately 5 million cells were implanted subcutaneously into the hind flank of female athymic nude mice (6 per group). Compound dosing began on day 21 when tumors reached an average size of 300–400 mm^3^. In the concurrent combination (), emibetuzumab was dosed for 7 cycles, with merestinib given for 50 days (dosing period is shown with the blue color double-arrowed horizontal bar). In the sequential combination () (dosing period and the sequence of the combination are shown with the red color double-arrowed horizontal bar), merestinib treatment was initially started as a single agent (Day 21), and upon tumor regression and regrowth to tumor size of 500 mm^3^, emibetuzumab was then added on for 6 cycles starting on Day 37. The last day of dosing was Day 70 and Day 78 for concurrent and sequential treatment group, respectively, and tumor growth was monitored post-treatment

Emibetuzumab was evaluated at both 10 mg/kg and 20 mg/kg once weekly dosing in the Hs746t xenograft model (Supplementary material, Fig. S2). At the end of 4 dosing cycles, there was no significant difference in anti-tumor effect between the two doses. The 10 mg/kg once weekly dose of emibetuzumab was used in the subsequent studies to address whether the combination of merestinib and emibetuzumab would lead to greater inhibition of Hs746t xenograft tumor growth. Concurrent combination treatments with merestinib and emibetuzumab were initiated when average tumor size reached 350 mm^3^ (Day 14) (Fig. 4a), and continued for 28 days. In the vehicle control group, 6 of the 7 mice were removed from the study by day 28 (after 14 day dosing) due to tumor burden (>2000mm^3^). Thus, statistical analysis of the anti-tumor effect of the treatments was performed on study day 27, although the dosing period continued through day 41. After 14 days of dosing, merestinib (6 mg/kg once daily) resulted in significant anti-tumor effect with T/C of 11.8% as compared to vehicle group (*p* < 0.001). As a single agent, emibetuzumab treatment (10 mg/kg once weekly) resulted in 38.6% tumor regression (p < 0.001). Concurrent combination of merestinib (6 mg/kg once daily) and emibetuzumab (10 mg/kg once weekly) resulted in 83.1% tumor regression (p < 0.001) and the combination was well tolerated. Single agent merestinib (12 mg/kg once daily) was shown previously (Fig. 3) to achieve 91.8% tumor regression. The concurrent combination of merestinib (12 mg/kg once daily) with emibetuzumab (10 mg/kg once daily) was well tolerated. This combination showed 91.2% tumor regression and was virtually identical to the 91.8% tumor regression with single agent merestinib at the 12 mg/kg dose. Thus the anti-tumor effect was achieved with the optimal dose of merestinib alone and not from the combination. The 83.1% tumor regression achieved with the suboptimal dose of merestinib (6 mg/kg once daily) in combination with emibetuzumab was statistically different than that achieved (91.2% tumor regression) with the optimal dose of merestinib (12 mg/kg once daily) in combination with emibetuzumab after 10 and 14 days of treatment (*p* = 0.011 and *p* = 0.022, respectively) (Fig. 4a). Concurrent combination of merestinib (6 mg/kg once daily) and emibetuzumab (10 mg/kg once weekly) resulted in 85% tumor regression after 28 days of dosing. While tumor outgrowth occurred upon termination of this combination treatment (Fig. 4a), the anti-tumor effect in the combination of merestinib (12 mg/kg once daily) with emibetuzumab (10 mg/kg once weekly) was durable, as no tumor growth was observed for 14 days after dosing termination.

Sequential combination treatment (single agent merestinib first followed by addition of emibetuzumab) was compared to the concurrent combination of merestinib (6 mg/kg once daily) with emibetuzumab (10 mg/kg once weekly) (Fig. 4b). In the sequential combination, merestinib dosing was initiated first when average tumor volume reached 300-400 mm^3^. Tumor regression was initially observed followed by outgrowth. Once tumors re-grew to 500mm^3^, emibetuzumab treatment was added to merestinib. Treatment in the concurrent combination cohort also commenced when average tumor volume reached 300-400 mm^3^. The sequential combination provided a longer treatment response than the concurrent combination treatment (Fig. 4b).

## Discussion

This is the first preclinical study reporting the evaluation of a MET-targeting kinase inhibitor in combination with an anti-MET antibody in a tumor model bearing MET exon 14 skipping. In this study, treatment with merestinib, a MET-targeting kinase inhibitor, at the once daily dose of 12 mg/kg resulted in durable tumor regression in mice bearing Hs746t xenograft tumors with *MET* exon14 skipping and *MET* amplification. The response was comparable to that of the type I MET inhibitor, crizotinib, dosed at 25 mg/kg once daily in this same model. The observed in vivo anti-tumor effect of merestinib and crizotinib is consistent with the potent in vitro anti-proliferative effect, inhibition of pMET and downstream signaling of the activated MET pathway in Hs746t cells. No evidence of acquired tumor resistance (tumor re-growth) to either single agent merestinib (12 mg/kg) or crizotinib was seen after 64 days of treatment. Treatment response in both groups was also durable, as no tumor growth was observed in mice 5 weeks after treatment termination.

The anti-MET antibody emibetuzumab showed much less in vitro anti-proliferative activity and pMET inhibition as compared to the MET kinase inhibitors evaluated. The extent of MET internalization by emibetuzumab was considerably less in Hs746t cells than in MKN45 cells. Both cell lines have *MET* amplification, but MKN45 cells bear no MET exon 14 skipping. This data would suggest that the degradation of MET internalized by emibetuzumab may be CBL-mediated. The in vitro reduction of total MET in Hs746t cells was enhanced when emibetuzumab was combined with merestinib, with about 50% reduction in cell surface MET receptor. In vivo anti-tumor effect in the Hs746t xenograft model with the MET-targeting antibody and kinase inhibitors reflects the same trend as the in vitro data in this MET exon 14 skipping cancer model. Hs746t xenografts treated with emibetuzumab showed a 7–10 day delay in tumor regression as compared to merestinib and crizotinib. Tumor regression was transient with emibetuzumab treatment and tumor outgrowth continued while on treatment.

The in vivo and in vitro data in this study consistently demonstrate that MET kinase inhibitors such as merestinib and crizotinib result in better anti-tumor response in the MET exon 14 skipping Hs746t model than the anti-MET antibody emibetuzumab. Similar in vitro and in vivo data in Hs746t were reported for another bivalent anti-MET antibody ABT-700. ABT-700 showed little or no effect in vitro in reducing total MET or pMET [21], while ABT-700 treatment of mice bearing Hs746t xenograft tumors resulted in either transient tumor stasis or slight tumor regression [21, 22]. Two other anti-MET antibodies, SAIT301 and DN30-Fab, were also evaluated in the Hs746t model [23, 24]. SAIT301, a bivalent MET antibody, was shown to internalize the MET receptor via a LRIG1-dependent, rather than a CBL-dependent mechanism [23]. In vitro treatment of the Hs746t cells with SAIT301 resulted in about 50% inhibition of cell proliferation and also about 50% reduction of the MET receptors. SAIT301 was further evaluated in vivo in the Hs746t model. Treatment was initiated when the average size of tumor was less than 100 mm^3^; the dosing period was only 14 days and the animals were sacrificed 4 days later. Tumor regression was observed during the 14 day dosing period. However, tumors were smaller when treatment was initiated, and without longer tumor monitoring, it is not known if the tumor regression observed was durable. The DN30-Fab is a MET antibody that induces the shedding of the extracellular domain of MET without MET agonistic activity [24]. DN30-Fab was able to down-regulate the levels of pMET in the Hs746t cells in vitro [24]. In vivo evaluation of DN30-Fab was studied in the Hs746t xenograft model with dosing initiated one day before the implant of the tumor cells [24]. In spite of the prophylactic treatment, DN30-Fab only delayed tumor growth. More recently, Sym-015, a cocktail of two bivalent MET IgG_1_ antibodies also did not show in vitro anti-proliferative activity in the Hs746t cells [25], but did show anti-tumor effect in the Hs746t xenograft model. The in vivo anti-tumor effect of Sym-015 was attributed to the antibody-dependent-cell-mediated cytotoxicity effect of the molecules. Thus, in this Hs746t preclinical model of MET exon 14 skipping, small molecule kinase inhibitors, including merestinib, showed more compelling anti-tumor effect than most anti-MET antibodies. The preclinical data from our study as well as from previous studies from others may provide explanation that to date, the published case reports of treatment response in cancer patients with MET exon 14 skipping were with MET-targeting small molecule kinase inhibitors, and not with MET-targeting antibody.

In vitro FACS data showed that a low concentration of merestinib (40 nM) was sufficient when combined with emibetuzumab (5 μg/ml, 33 nM) to enhance the reduction of total MET in Hs746t cells. Each single agent by itself did not induce the reduction of total MET. Increasing merestinib to 100 nM did not further enhance the reduction of total MET when combined with emibetuzumab. These data are consistent with the in vivo findings of concurrent low dose merestinib (suboptimal at 6 mg/mg once daily) combined with emibetuzumab, showing enhanced anti-tumor effect over each single agent at their respective dose.

While single agent merestinib (12 mg/kg once daily) proved to be highly efficacious in the Hs746t xenograft model, with no further benefit observed with the addition of emibetuzumab, clinical evaluation of the combination may still be warranted. Preclinical data may or may not be entirely translatable to patients with tumors bearing MET exon 14 skipping. Data from this study showed that sequential treatment combination of merestinib (6 mg/kg once daily) with emibetuzumab provided more durable antitumor effect than concurrent combination (Fig. 4b). In the sequential combination, when tumors stopped responding to the suboptimal dose of merestinib, the addition of emibetuzumab resulted in a sustained anti-tumor response, which could provide a relevant treatment option to patients in the clinical setting. Thus far, published case reports have only described treatment response with single agent MET-targeting kinase inhibitor in cancer patients with tumors bearing MET exon 14 skipping.

Crizotinib is a type I MET kinase inhibitor. Several case studies of cancer patients with MET exon 14 skipping or MET amplification who progressed on crizotinib treatment had either pre-existing or acquired secondary point mutation(s) in the MET kinase domain [26–30]. These reported point mutations are in residues D1228 and Y1230. Merestinib, a type II MET kinase inhibitor, was shown to retain potency in vitro for these reported pre-existing or acquired MET kinase domain point mutations [14, 28]. These preclinical data support the clinical evaluation of merestinib in the treatment of naïve patients with MET exon 14 skipping with or without *MET* amplification and in patients who progressed on a type I MET kinase inhibitor (NCT02920996). The data from this first reported preclinical study of the combination of merestinib, a MET kinase inhibitor with emibetuzumab, an anti-MET antibody, also support clinical evaluation of sequential combination treatment of single agent merestinib first followed by the addition of emibetuzumab when patients progress on merestinib.

## Electronic supplementary material

ESM 1 (DOCX 99 kb)
