# Supplementary material for: MET-targeting antibody (emibetuzumab) and kinase inhibitor (merestinib) as single agent or in combination in a cancer model bearing MET exon 14 skipping
Source: Invest New Drugs. 2017 Nov 29;36(4):536–44. doi: 10.1007/s10637-017-0545-x (PMC6061111; doi:10.1007/s10637-017-0545-x)
Supplement: Supplementary file 1 — (DOCX 99 kb) [file 10637_2017_545_MOESM1_ESM.docx]

**Fig. S1** *In vitro* effect of emibetuzumab, merestinib or the combination on cell surface MET receptors in Hs746t cells.

(a) Effect of emibetuzumab on MET receptor internalization in Hs746t cells. Cells were treated overnight at 37°C with 33nM (5µg/ml) emibetuzumab, 67nM (5µg/ml) humanized one-armed 5D5 (OA-5D5), or 33nM (5µg/ml) hIgG_4_ isotype control, dissociated with enzyme-free dissociation solution, and stained with Alexa-488 labeled MET detection antibody (that recognizes a separate MET epitope from emibetuzumab and OA-5D5), and measured by FACS for remaining cell surface MET. 10,000 events were acquired by FACS for each sample. Results are shown as the mean ± SE and are pooled from two independent experiments. * p< 0.01, emibetuzumab vs. hIgG_4_.

(b) Effect of combination of emibetuzumab and merestinib on MET receptor internalization. Hs746t cells were treated overnight at 37°C with 33nM emibetuzumab or hIgG_4_ isotype control, 40nM or 100nM merestinib or the combinations, dissociated with enzyme-free dissociation solution, and stained with Alexa-488 labeled MET detection antibody (that recognizes a separate MET epitope from emibetuzumab), and measured by FACS for remaining cell surface MET. 10,000 events were acquired by FACS for each sample. Results are shown as the mean ± SE and are pooled from two independent experiments. * p< 0.05, emibetuzumab vs. combinations.

**(a) (b)**

**
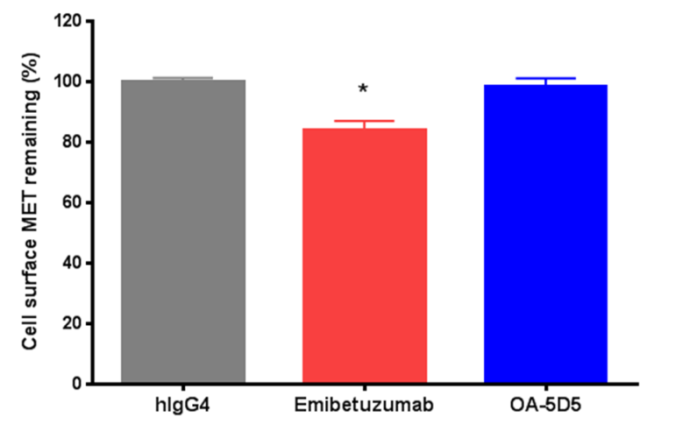

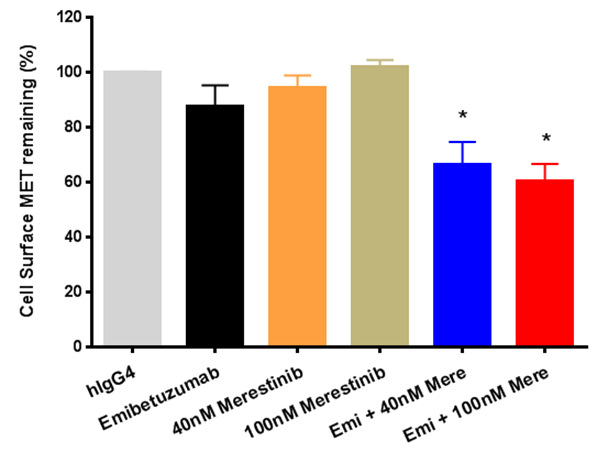
**

**Fig. S2** Comparing two doses of emibetuzumab *in vivo* in the Hs746t-derived xenograft model.

Approximately 5 x 10^6^ Hs746t cells were implanted subcutaneously into the hind flank of the female athymic nude mice (6 per group). Emibetuzumab was administered when tumors reached an average size of 300-400 mm^3^. Emibetuzumab was administered via intraperitoneal injection once every 7 days, at 10 or 20 mg/kg for 4 cycles. When the final tumor measurement was taken (Day 51), tumor volumes from the two groups were not statistically different from each other (p=0.129).
